# Supplementary material for: The DEAD-box ATPase Dbp10/DDX54 initiates peptidyl transferase center formation during 60S ribosome biogenesis
Source: Nat Commun. 2024 Apr 17;15:3296. doi: 10.1038/s41467-024-47616-7 (PMC11024185; doi:10.1038/s41467-024-47616-7)
Supplement: Supplementary file 1 — Supplementary Information [file 41467_2024_47616_MOESM1_ESM.pdf]

Supplementary Information

The DEAD-box ATPase Dbp10/DDX54 initiates peptidyl transferase center formation during 60S ribosome biogenesis

Victor E. Cruz[1#], Christine S. Weirich[1], Nagesh Peddada[1†] and Jan P. Erzberger[1*]

[1]Department of Biophysics, UT Southwestern Medical Center - ND10.124B, 5323 Harry Hines Blvd., Dallas, TX 75390, USA
*Corresponding author. Email: jan.erzberger@utsouthwestern.edu

# present address: O'Donnell Brain Institute/CAND, UT Southwestern Medical Center, 5323 Harry Hines Blvd., Dallas, TX 75390, USA

† present address: Center for the Genetics of Host Defense, UT Southwestern Medical Center, 5323 Harry Hines Blvd., Dallas, TX 75390, USA

Supplementary Table 1 | Yeast strains used in this study

Supplementary Table 2 | Plasmids used in this study

Supplementary Table 3 |Cryo-EM data collection, refinement, and validation statistics

Supplementary Figure 1 | Purification of pre-60S intermediates.

Supplementary Figure 2 | Micrographs, 2D classes, and 3D classification scheme for the pre-catalysis dataset.

Supplementary Figure 3 | Fourier shell correlation (FSC) curves and local resolution plots for the pre-catalytic intermediate.

Supplementary Figure 4 | Micrographs, 2D classes, and 3D classification scheme for catalytic intermediate dataset.

Supplementary Figure 5|Fourier shell correlation (FSC) curves and local resolution plots for the catalytic intermediate.

Supplementary Figure 6 | Micrographs, 2D classes, and 3D classification scheme for post-catalysis dataset.

Supplementary Figure 7 | Fourier shell correlation (FSC) curves and local resolution plots for the catalytic intermediate.

Supplementary Figure 8 | Comparison of Dbp10/DDX54 structures and sequence conservation within the PTC.

Supplementary Table 1 | Yeast strains used in this study

Strain	Relevant genotype	Source
BY4741	*MATa his3⊿1 leu2⊿0 met15⊿0 ura3⊿0*	[1]
YJE190	*MATa his3⊿1 leu2⊿0 met15⊿0 ura3⊿0::sfGFP-3xSTREP-bdNEDD8-MYC-dbp10R522V::URA3 trp1Δ::*P_{ACT1}*-LexA-ER-haB112-TRP1*	This paper
YJE205	*MATa his3⊿1 leu2⊿0 met15⊿0 ura3⊿0::sfGFP-3xSTREP-bdNEDD8-MYC- DBP10::URA3 trp1Δ::pACT1-LexA-ER-haB112-TRP1*	This paper
YJE231	*MATa his3⊿1 leu2⊿0 met15⊿0 ura3⊿0::sfGFP-3xSTREP-bdNEDD8-MYC- -dbp10*^{R522V} *URA3 trp1Δ::*P_{ACT1}*-LexA-ER-haB112-TRP1 BRX1-3xFLAG-PrtA::HygMX*	This paper
YJE382	*MATa his3⊿1 leu2⊿0 met15⊿0 ura3⊿0::sfGFP-3xSTREP-bdNEDD8-MYC- -dbp10*^{R522V}*::URA3 trp1Δ::PACT1-LexA-ER-haB112-TRP1 SSF1-3xFLAG-2xPrtA::hphMX6 nsa1Δ417-438::NrsR*	This paper
YJE484	*MATa his3⊿1 leu2⊿0 ura3⊿0 dbp10Δ::HygMX pURA-DBP10*	This paper
YJE552	*MATa his3⊿1 leu2⊿0 met15⊿0 ura3⊿0::sfGFP-3xSTREP-bdNEDD8-MYC-dbp10K187A::URA3 trp1Δ::* P_{ACT1}*-LexA-ER-haB112-TRP1*	This paper
YJE554	*MATa his3⊿1 leu2⊿0 met15⊿0 ura3⊿0::sfGFP-3xSTREP-bdNEDD8-MYC- dbp10D288A::URA3 trp1Δ::* P_{ACT1}*-LexA-ER-haB112-TRP1*	This paper
YJE781	*MATa his3⊿1 leu2⊿0::osTIR1::LEU2 met15⊿0 ura3⊿0 trp1Δ::PACT1-LexA-ER-haB112-TRP1 NOC2-3X-FLAG-2XPrtA::hphMX6 SPB4-IAA17::NrsR dbp10Δ::HIS3-2xStrep-bdNEDD8-DBP10*	This paper
YJE1243	*MATa his3⊿1 leu2⊿0 met15⊿0 ura3⊿0 trp1Δ::*P_{ACT1}*-LexA-ER-haB112-TRP1 SSF1-3xFLAG-2xPrtA::hphMX6 dbp10Δ::HIS3-2xStrep-bdNEDD8-DBP10*	This paper
YJE1277	*MATa his3⊿1 leu2⊿0 ura3⊿0::sfGFP-3xSTREP-bdNEDD8-MYC-DBP10::URA3 BRX1-3xFLAG-PrtA::HygMX*	This paper

Supplementary Table 2 | Plasmids used in this study

Plasmid	Description	Source
pRS313	pCEN-HIS	ATCC
pRS315	pCEN-LEU	ATCC
pJE664	pRS406-4xLexA-box-$P_{minCYC1}$-sfGFP-3xstrep-Tag II-DBP10-Myc-T_{CYC1}	This paper
pJE727	pBluescript-DBP10	This paper
pJE728	pRS316-DBP10	This paper
pJE729	pRS315-DBP10	This paper
pJE748	pRS315-dbp10^{K187A}	This paper
pJE749	pRS315-dbp10^{K187R}	This paper
pJE750	pRS315-dbp10^{D288A}	This paper
pJE751	pRS315-dbp10^{E289A}	This paper
pJE753	pRS315-dbp10^{R522V}	This paper
pJE788	pRS315-dbp10$^{\Delta 561-665}$	This paper
pJE792	pRS315-dbp10$^{\Delta 667-631}$	This paper
pJE815	pRS406-4xLexA-box-$P_{minCYC1}$-sfGFP-3xstrep-bdNEDD8-MYC -dbp10^{K187A}-T_{CYC1}	This paper
pJE817	pRS406-4xLexA-box-$P_{minCYC1}$-sfGFP-3xstrep-bdNEDD8-MYC-dbp10^{D288A}-T_{CYC1}	This paper
pJE821	pRS406-4xLexA-box-$P_{minCYC1}$-sfGFP-3xstrep-bdNEDD8-MYC-dbp10^{R522V}-T_{CYC1}	This paper
pJE1218	pRS313-DBP10	This paper
pJE1222	pRS313-dbp10$^{\Delta nop2int}$	This paper
pJE1384	pRS313-dbp10$^{1-958}$	This paper
pJE1386	pRS313-dbp10$^{1-911}$	This paper
pJE1388	pRS313-dbp10$^{\Delta 926-943}$	This paper
pJE1390	pRS313-dbp10^{AAAA}	This paper

Supplementary Table 3 | Cryo-EM data collection, refinement, and validation statistics

	Pre-catalysis state	Catalysis state	Post-catalysis state
**Data collection and processing**			
Magnification	81,000x	81,000x	81,000x
Voltage (kV)	300	300	300
Electron exposure (e–/Å^2)	45	65.5	65.5
Defocus range (μm)	-0.9 – (-2.2)	-0.9 – (-2.2)	-0.9 – (-2.2)
Pixel size (Å)	1.059	1.08	1.08
Symmetry imposed	C1	C1	C1
Initial particle images (no.)	2,374,356	3,848,428	1,202,056
Final particle images (no.)	374,572	192,741	175,421
Map resolution (Å)	2.53	2.7	2.66
FSC threshold	0.143	0.143	0.143
Map resolution range (Å)	2.118 – 10	2.16 – 10	2.16 – 10
**Refinement**			
Model resolution (Å)	2.69	2.91	2.77
FSC threshold	0.5	0.5	0.5
Map sharpening *B* factor (Å^2)	74	68.01	79.5
Model composition			
Non-hydrogen atoms	119220	121247	152502
Protein residues	8913	9280	11563
Nucleotides	2223	2178	2815
Ligands	2	2	6
B factors (Å^2)			
Protein	30.71	31.67	16.77
Nucleotides	70.17	57.83	23.53
Ligand	65.13	38.48	65.46
R.m.s. deviations			
Bond lengths (Å)	0.002	0.002	0.003
Bond angles (°)	0.450	0.497	0.501
Validation			
MolProbity score	1.19	1.64	1.34
Clashscore	4.07	6.49	5.41
Poor rotamers (%)	0.51	0.59	0.64
Ramachandran plot			
Favored (%)	98.14	98.02	98.09
Allowed (%)	1.86	1.98	1.91
Disallowed (%)	0	0	0

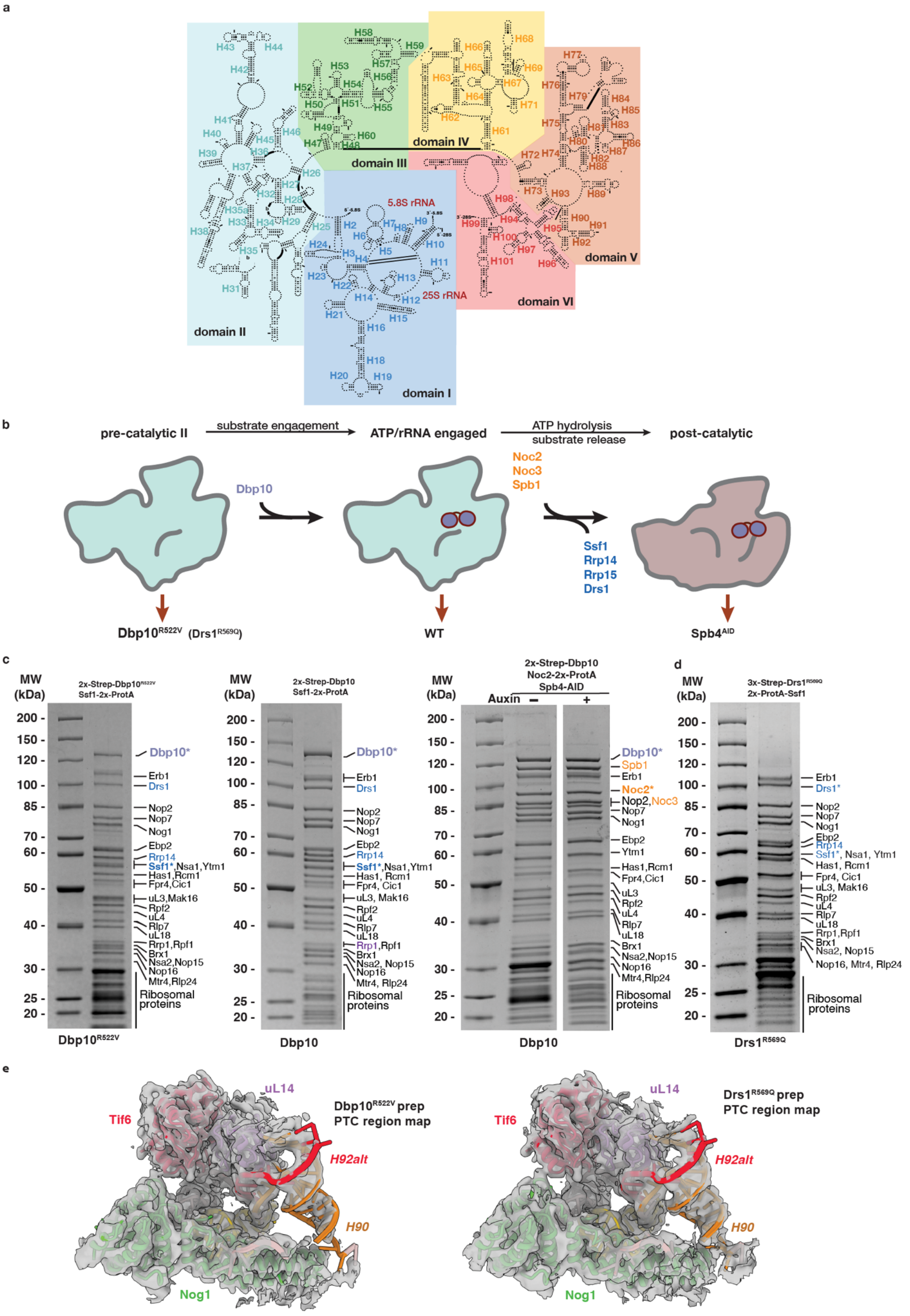

a
domain I
domain II
domain III
domain IV
domain V
domain VI
5.8S rRNA
25S rRNA
H2 H3 H4 H5 H6 H7 H8 H9 H10 H11 H12 H13 H14 H15 H16 H18 H19 H20 H21 H22 H23 H24 H25
H26 H27 H28 H29 H31 H32 H33 H34 H35 H35a H36 H37 H38 H39 H40 H41 H42 H43 H44 H45 H46
H47 H48 H49 H50 H51 H52 H53 H54 H55 H56 H57 H58 H59 H60
H61 H62 H63 H64 H65 H66 H67 H68 H69 H71
H72 H73 H74 H75 H76 H77 H79 H80 H81 H82 H83 H84 H85 H86 H87 H88 H89 H90 H91 H92 H93
H94 H95 H96 H97 H98 H99 H100 H101
b
pre-catalytic II
substrate engagement
ATP/rRNA engaged
ATP hydrolysis
substrate release
post-catalytic
Dbp10
Noc2
Noc3
Spb1
Ssf1
Rrp14
Rrp15
Drs1
Dbp10R522V (Drs1R569Q)
WT
Spb4AID
c
MW (kDa)
2x-Strep-Dbp10R522V
Ssf1-2x-ProtA
2x-Strep-Dbp10
Ssf1-2x-ProtA
2x-Strep-Dbp10
Noc2-2x-ProtA
Spb4-AID
Auxin − +
200 150 120 100 85 70 60 50 40 30 25 20
Dbp10*
Spb1
Erb1
Drs1
Noc2*
Nop2,Noc3
Nop2
Nop7
Nog1
Ebp2
Ytm1
Rrp14
Ssf1*,Nsa1,Ytm1
Has1,Rcm1
Fpr4,Cic1
uL3,Mak16
Rpf2
uL4
Rlp7
uL18
Rrp1,Rpf1
Brx1
Nsa2,Nop15
Nop16
Mtr4,Rlp24
Ribosomal proteins
Dbp10R522V
Dbp10
d
3x-Strep-Drs1R569Q
2x-ProtA-Ssf1
Drs1*
Ssf1*, Nsa1, Ytm1
Nop16, Mtr4, Rlp24
Drs1R569Q
e
Tif6
uL14
Dbp10R522V prep
PTC region map
Drs1R569Q prep
PTC region map
H92alt
H90
Nog1

Supplementary Figure 1 | Purification of pre-60S intermediates. a. Schematic of the mature 25S/5.8S rRNA secondary structure, colored to highlight rRNA domains. Individual helices in each domain are labeled. **b.** Flowchart and cartoon model of nucleolar pre-60S intermediates characterized in this study. (left-cyan) *Dbp10*^{R522V} dissociates from our sample and is used as a pre-catalytic mimic. (middle-cyan) the catalytic intermediate was obtained from wild-type cells purified in the presence of BeF_3 (right). The post-catalytic state is characterized by a concerted exchange of RBFs denoted in the flowchart and colored as in Fig.1. **c.** Coomassie-stained 4−20% SDS−PAGE gels of purified pre-60S intermediates (indicated by red arrows). Individual bands are labeled and proteins that are part of the RBF exchange are highlighted as in a. Affinity labeled proteins are marked with an asterisk. Protein identification was determined based on molecular weight, ambiguous bands were identified by mass spectrometry. For the post-catalytic intermediate, auxin-induced degradation of Spb4 resulted in ~10-fold enrichment of Dbp10•Noc2 pre-60S. **d.** Coomassie-stained 4−20% SDS−PAGE of intermediates purified from a *drs1*^{R569Q} strain. These intermediates are compositionally identical to the intermediates from the *dbp10*^{R522V} strain, except for the absence of Dbp10. **e.** The reconstructed PTC region of the intermediates obtained from the *dbp10*^{R522V} and *drs1*^{R569Q} strains are near identical (maps low-pass filtered to 4.5Å), showing that the structure we observe in our pre-catalysis reconstruction is already present before Dbp10 binding.

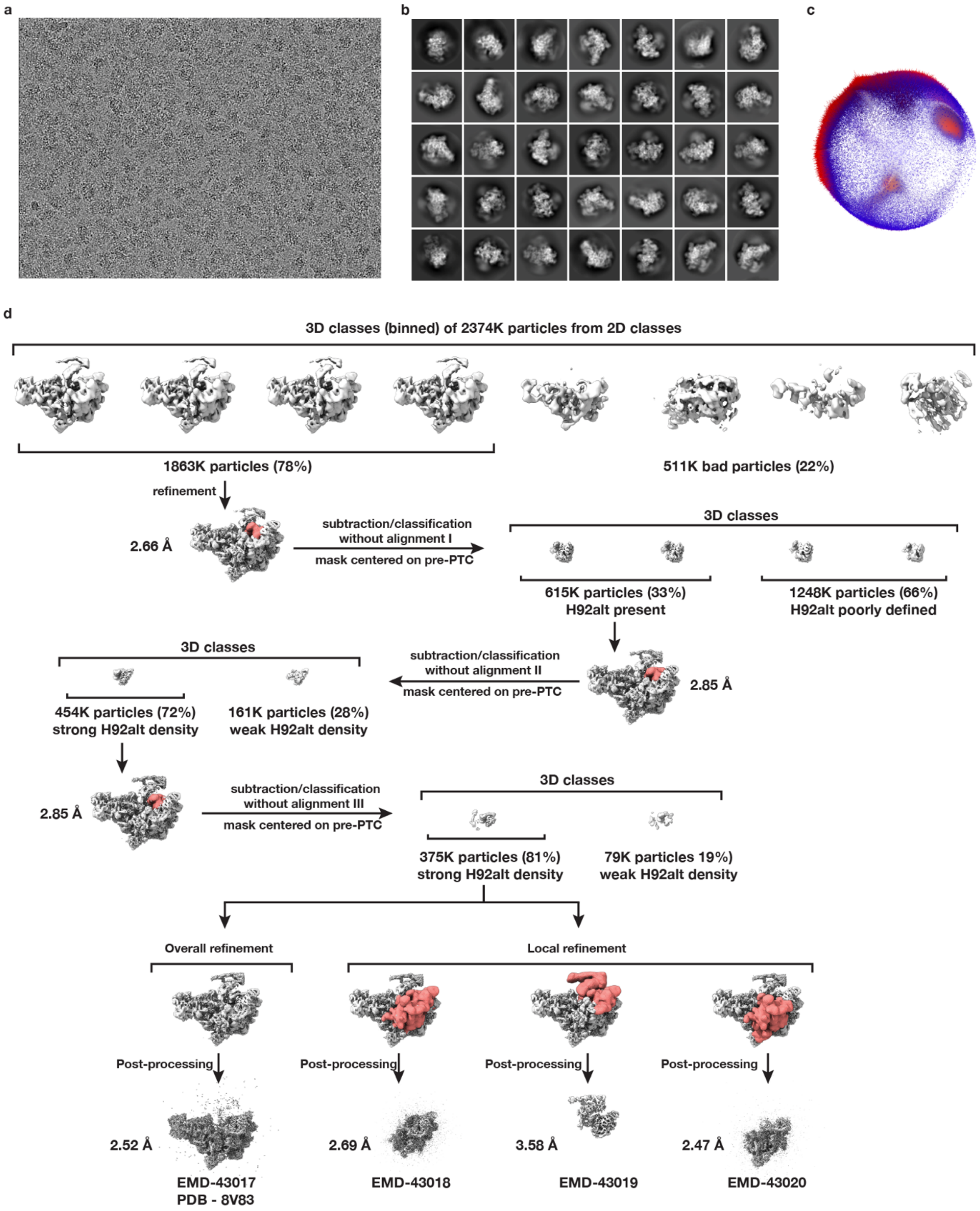

a
b
c
d
3D classes (binned) of 2374K particles from 2D classes
1863K particles (78%)
511K bad particles (22%)
refinement
2.66 Å
subtraction/classification
without alignment I
mask centered on pre-PTC
3D classes
615K particles (33%)
H92alt present
1248K particles (66%)
H92alt poorly defined
3D classes
subtraction/classification
without alignment II
mask centered on pre-PTC
2.85 Å
454K particles (72%)
strong H92alt density
161K particles (28%)
weak H92alt density
2.85 Å
subtraction/classification
without alignment III
mask centered on pre-PTC
3D classes
375K particles (81%)
strong H92alt density
79K particles 19%)
weak H92alt density
Overall refinement
Local refinement
Post-processing
Post-processing
Post-processing
Post-processing
2.52 Å
2.69 Å
3.58 Å
2.47 Å
EMD-43017
PDB - 8V83
EMD-43018
EMD-43019
EMD-43020

Supplementary Figure 2 | Micrographs, 2D classes, and 3D classification scheme for the pre-catalysis dataset. a. Representative micrograph out of a total of 10539 obtained from $Dbp10^{R522V}$•Ssf1 samples. **b.** Representative 2D classes with a broad distribution of orientations showing clear secondary structure features were selected for further processing and resulted in a total of 2734K particles. **c.** Angular distribution of particles for the final refinement. **d.** Workflow of the 3D classification scheme. Map volumes are shown for all steps and masks (red) indicate volume selected for local classification. Major sorting and classification criteria, particle numbers and percentages (for each 3D classification) as well as final map resolutions are indicated. Optimization of the T-factor was critical during 3D classification performed in Relion 4.0, respectively during the three rounds of local skip-align classification T-factors of 60, 60, and 40 were used. Mask selection for local classification and refinement were adjusted at each juncture using the quality of local maps as a metric to guide parameter optimization.

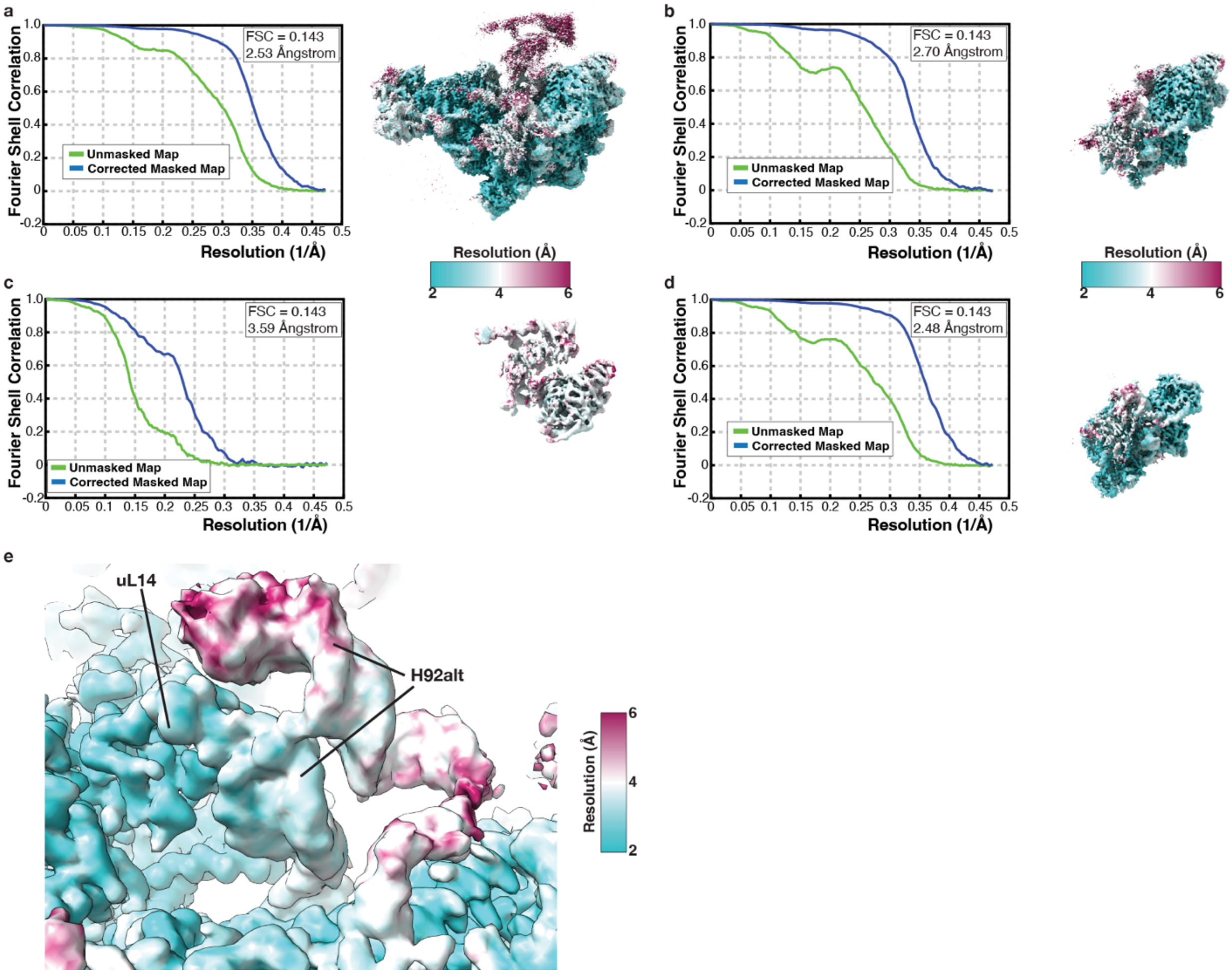

Supplementary Figure 3 | Fourier shell correlation (FSC) curves and local resolution plots for the pre-catalytic intermediate. (Left) Fourier shell correlation plots and (Right) maps colored according to local resolution estimates for **a.** Overall map, **b.** Locally refined map focused on the PTC, **c.** Locally refined map focused on the PTC and the L1 stalk, **d.** Locally refined map focused on the Rrp14, Rrp15, Ssf1 trimeric complex. **e.** Detail of the local resolution distribution around the premature PTC structure. The cryo-EM density for H92alt and uL14 is labeled.

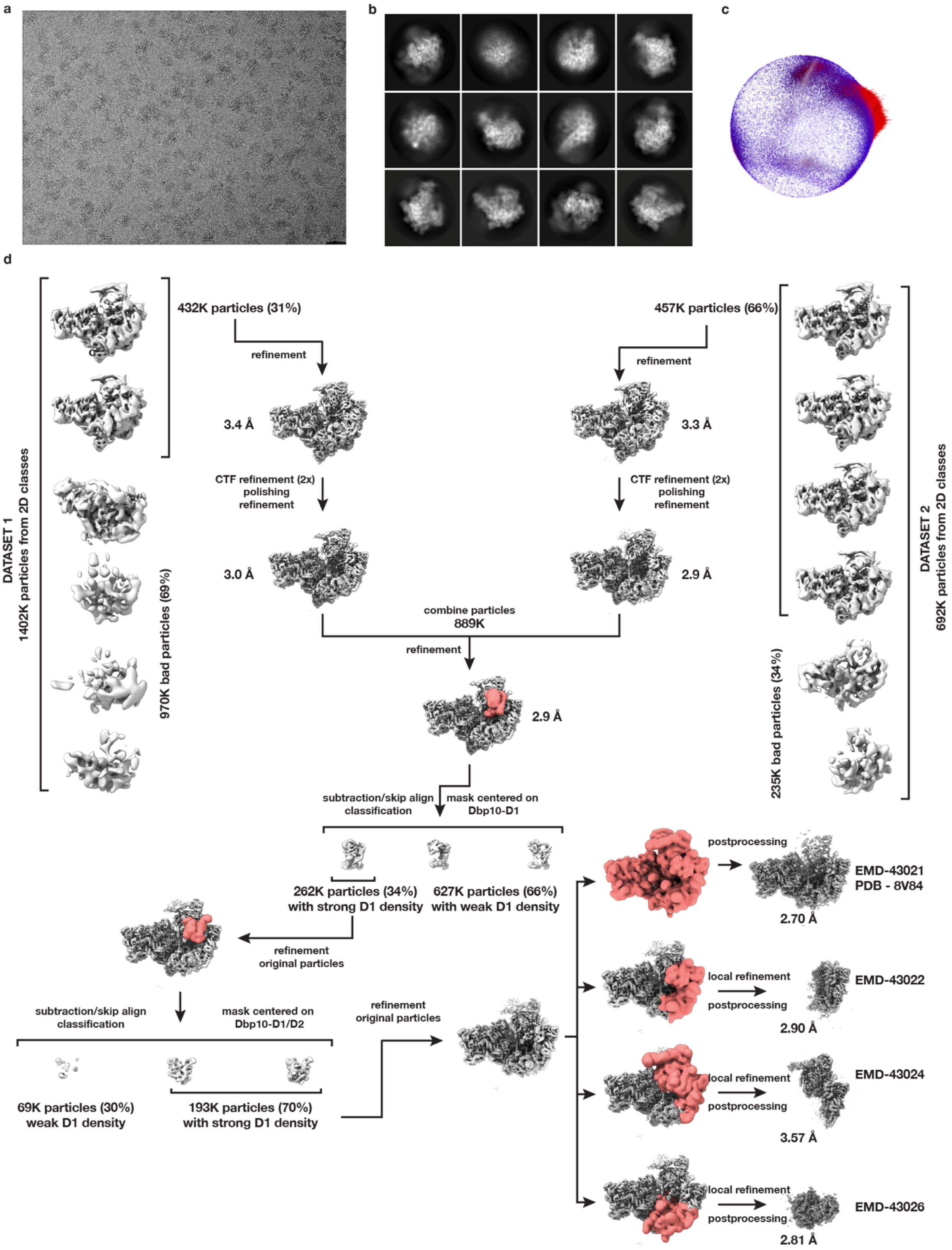

a
b
c
d
DATASET 1
1402K particles from 2D classes
970K bad particles (69%)
432K particles (31%)
refinement
3.4 Å
CTF refinement (2x)
polishing
refinement
3.0 Å
457K particles (66%)
refinement
3.3 Å
CTF refinement (2x)
polishing
refinement
2.9 Å
DATASET 2
692K particles from 2D classes
235K bad particles (34%)
combine particles
889K
refinement
2.9 Å
subtraction/skip align
classification
mask centered on
Dbp10-D1
262K particles (34%)
with strong D1 density
627K particles (66%)
with weak D1 density
refinement
original particles
subtraction/skip align
classification
mask centered on
Dbp10-D1/D2
69K particles (30%)
weak D1 density
193K particles (70%)
with strong D1 density
refinement
original particles
postprocessing
EMD-43021
PDB - 8V84
2.70 Å
local refinement
postprocessing
EMD-43022
2.90 Å
local refinement
postprocessing
EMD-43024
3.57 Å
local refinement
postprocessing
EMD-43026
2.81 Å

Supplementary Figure 4 | Micrographs, 2D classes, and 3D classification scheme for catalytic intermediate dataset. a. Representative micrograph out of a total of 20054 obtained from Dbp10•Ssf1 samples. Two datasets obtained from the identical sample on two separate grids were combined from this analysis. **b.** Representative 2D classes with a broad distribution of orientations showing clear secondary structure features were selected for further processing and resulted in a total of 2094K particles. **c.** Angular distribution of particles for the final refinement. **d.** Workflow of the 3D classification scheme. Map volumes are shown for all steps and masks (red) indicate volume selected for local classification. Major sorting and classification criteria, particle numbers and percentages (for each 3D classification) as well as final map resolutions are indicated. Particles from the two datasets were separately 3D and CTF refined, polished, and combined subsequently during 3D refinement resulting in 889K particles. Mask selection for local classification and refinement were adjusted around Dbp10-D1 with T-factors of 60 and 40 respectively for the first and second selection rounds, at each juncture the quality of the resulting local maps guided parameter optimization.

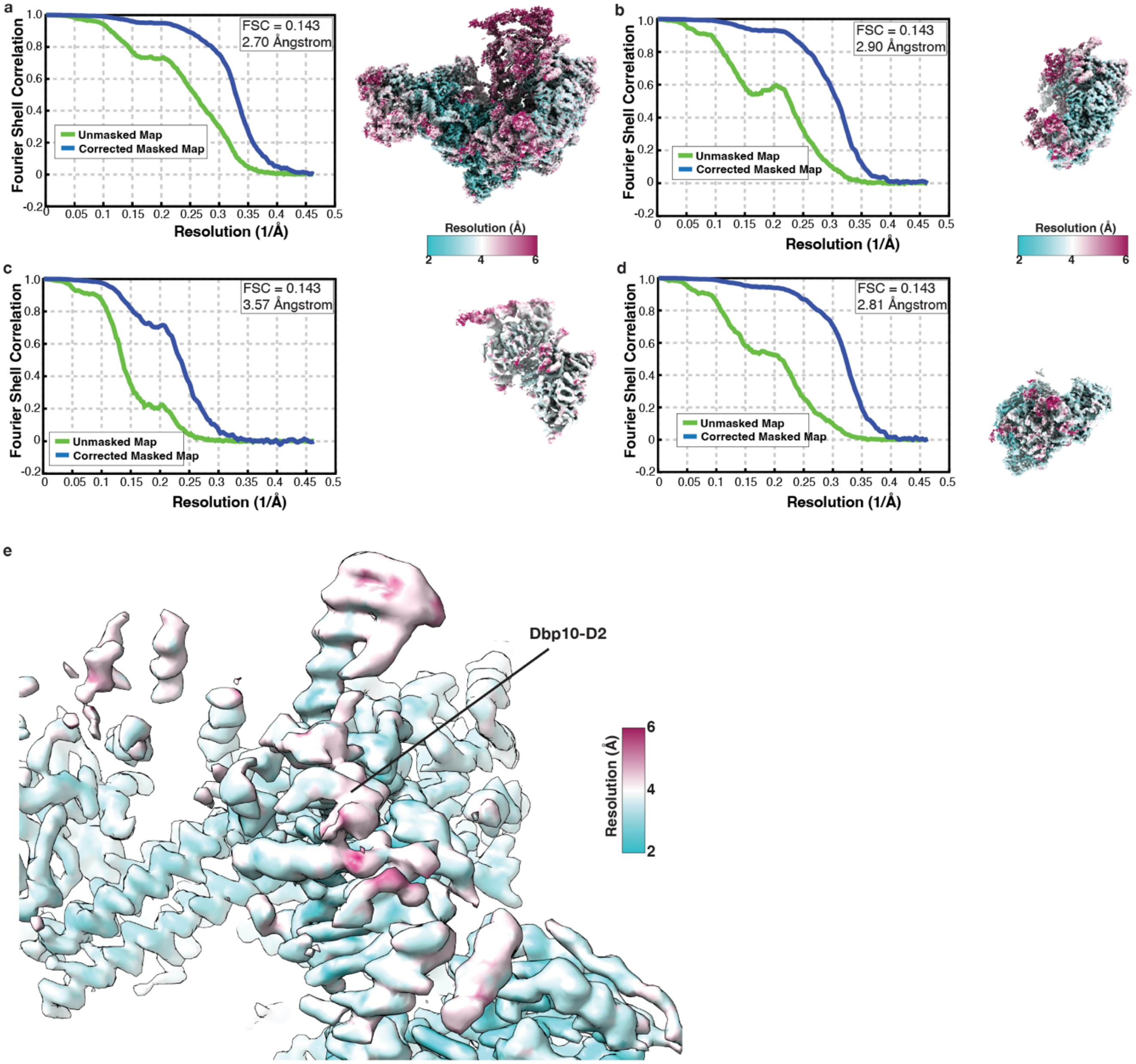

Supplementary Figure 5 | Fourier shell correlation (FSC) curves and local resolution plots for the catalytic intermediate. (Left) Fourier shell correlation plots and (Right) maps colored according to local resolution estimates for **a.** Overall map, **b.** Locally refined map focused on the Dbp10, **c.** Locally refined map focused on the L1 stalk, **d.** Locally refined map focused on the Rrp14, Rrp15, Ssf1 trimeric complex. **e.** Detail of the local resolution distribution around the Dbp10 binding site. The cryo-EM density corresponding to Dbp10-D2 is labeled.

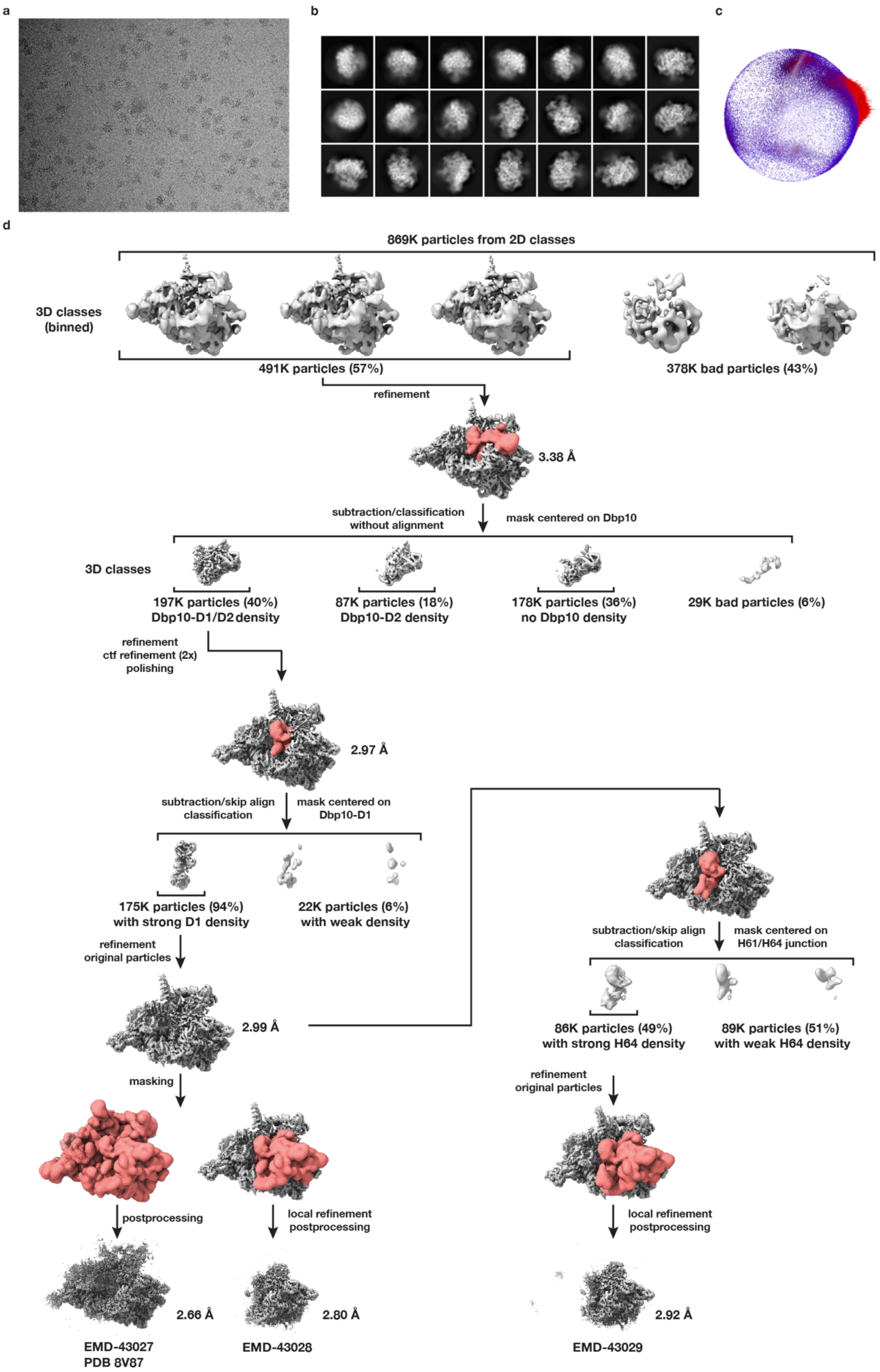
a
b
c
d
869K particles from 2D classes
3D classes (binned)
491K particles (57%)
378K bad particles (43%)
refinement
3.38 Å
subtraction/classification without alignment
mask centered on Dbp10
3D classes
197K particles (40%) Dbp10-D1/D2 density
87K particles (18%) Dbp10-D2 density
178K particles (36%) no Dbp10 density
29K bad particles (6%)
refinement
ctf refinement (2x)
polishing
2.97 Å
subtraction/skip align classification
mask centered on Dbp10-D1
175K particles (94%) with strong D1 density
22K particles (6%) with weak density
refinement original particles
2.99 Å
subtraction/skip align classification
mask centered on H61/H64 junction
86K particles (49%) with strong H64 density
89K particles (51%) with weak H64 density
refinement original particles
masking
postprocessing
local refinement postprocessing
local refinement postprocessing
2.66 Å
2.80 Å
2.92 Å
EMD-43027
PDB 8V87
EMD-43028
EMD-43029

Supplementary Figure 6 | Micrographs, 2D classes, and 3D classification scheme for post-catalysis dataset. a. Representative micrograph out of a total of 8882 obtained from Dbp10•Noc2 using Spb4-depleted samples. **b.** Representative 2D classes with a broad distribution of orientations showing clear secondary structure features were selected for further processing and resulted in a total of 869K particles. **c.** Angular distribution of particles for the final refinement. **d.** Workflow of the 3D classification scheme. Map volumes are shown for all steps and masks (red) indicate volume selected for local classification and 3D refinement. Major sorting and classification criteria, particle numbers and percentages (for each 3D classification step) as well as final map resolutions are indicated. Mask selection for local classification was initially centered on Dbp10 with a T-factor of 60 and subsequently on Dbp10-D1 using a T-factor of 40, at each juncture the quality of the resulting local maps guided parameter optimization.

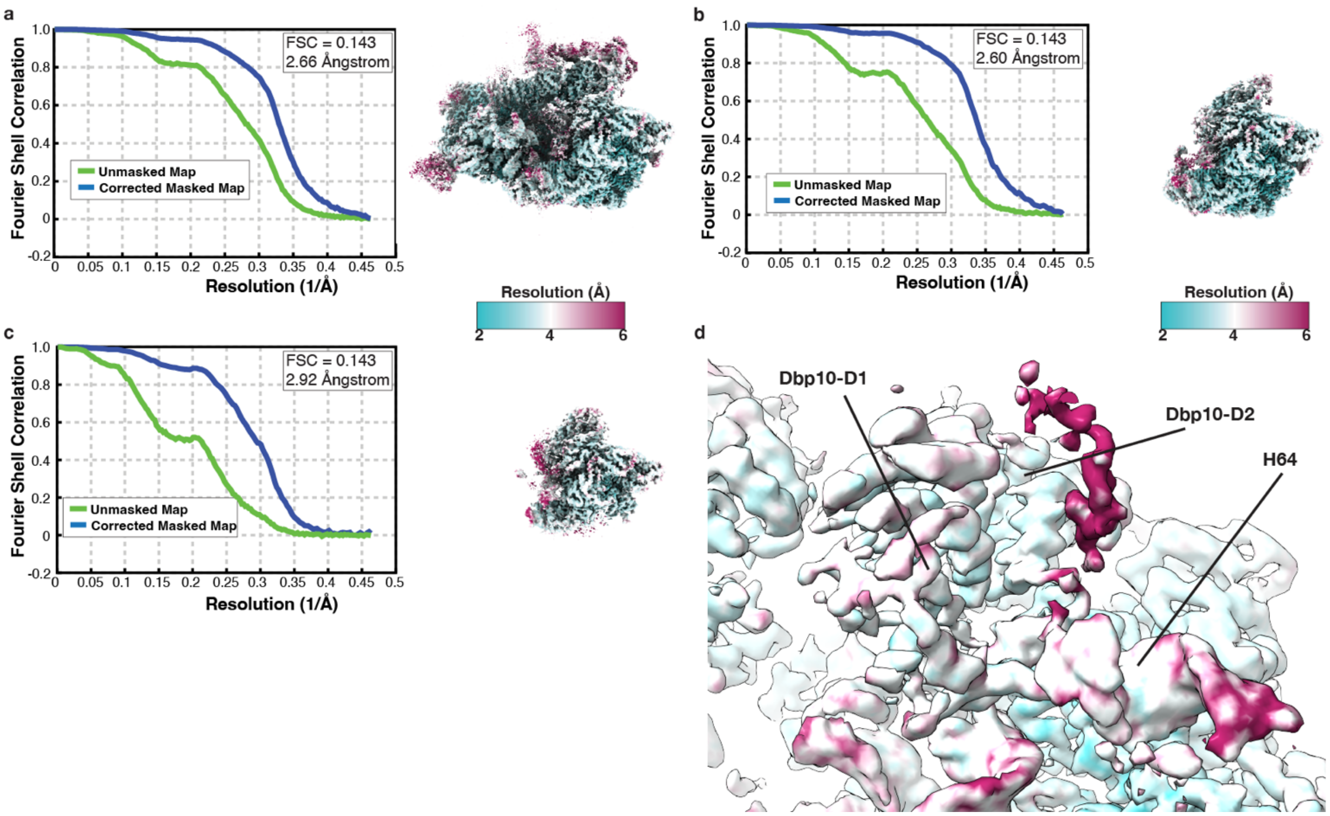
a
b
c
d
FSC = 0.143
2.66 Ångstrom
2.60 Ångstrom
2.92 Ångstrom
Fourier Shell Correlation
Resolution (1/Å)
Unmasked Map
Corrected Masked Map
Resolution (Å)
2
4
6
Dbp10-D1
Dbp10-D2
H64

Supplementary Figure 7 | Fourier shell correlation (FSC) curves and local resolution plots for the catalytic intermediate. (Left) Fourier shell correlation plots and (Right) maps colored according to local resolution estimates for **a.** Overall map, **b.** Locally refined map focused around Dbp10, **c.** Locally refined map of the subset with improved density for H61/H64. **d.** Detail of the local resolution distribution around the Dbp10 binding site. The cryo-EM density corresponding to Dbp10-D1 and Dbp10-D2 is labeled.

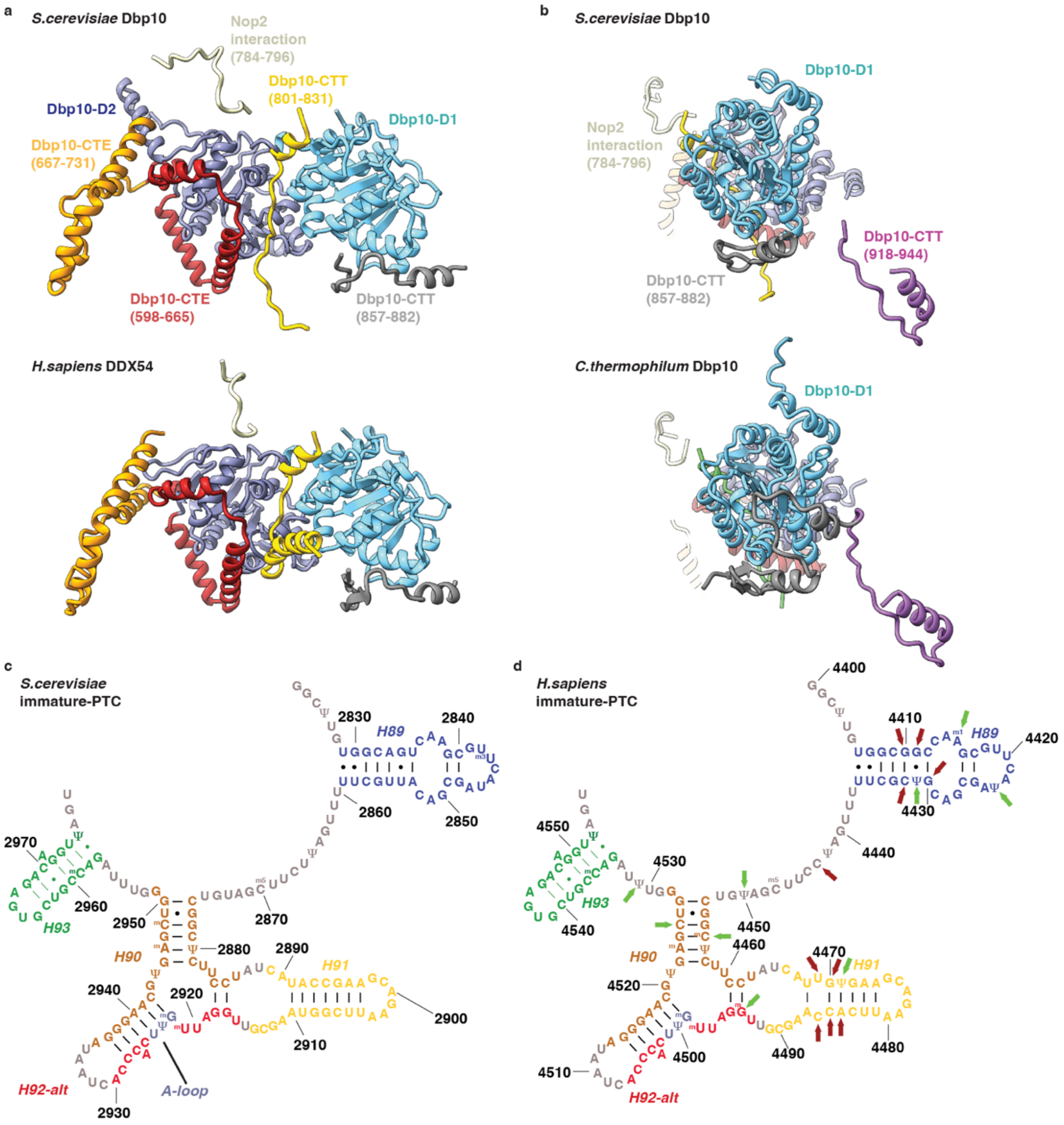
a
S.cerevisiae Dbp10
Nop2 interaction (784-796)
Dbp10-CTT (801-831)
Dbp10-D2
Dbp10-D1
Dbp10-CTE (667-731)
Dbp10-CTE (598-665)
Dbp10-CTT (857-882)
H.sapiens DDX54
b
S.cerevisiae Dbp10
Dbp10-D1
Nop2 interaction (784-796)
Dbp10-CTT (918-944)
Dbp10-CTT (857-882)
C.thermophilum Dbp10
Dbp10-D1
c
S.cerevisiae immature-PTC
H89
H93
H90
H91
H92-alt
A-loop
d
H.sapiens immature-PTC
H89
H93
H90
H91
H92-alt

Supplementary Figure 8 | Comparison of Dbp10/DDX54 structures and sequence conservation within the PTC. a. Cartoon structures of *S.cerevisae* Dbp10 (top) and *H.sapiens* DDX54 (bottom – PDB 8I9X[20]). Conserved structural elements within Dbp10/DDX54 are individually colored and labeled in the *S.cerevisiae* structure. **b.** Cartoon structures of *S.cerevisiae* (top) and *C.thermophilum* (bottom-PDB 8FKY[21]) Dbp10 rotated 90° compared to panel a. The most distal CTT component is conserved between these organisms. The segment spanning the domain interface of *C.thermophilum* Dbp10 (green) was modeled as a section of the N-terminal tail of Dbp10. We believe this is a misinterpretation of the experimental density. **c.** Secondary structure diagram of the *S.cerevisiae* premature PTC structure. **d.** The same secondary structure imposed on the *H.sapiens* rRNA sequence to form a hypothetical immature PTC structure. Positions with variations in either base identity (red arrows) or modifications (green arrows) are indicated. All nucleotides involved in H92alt and H90/H92 formation are invariant between the two species.

Supplementary References

1 Brachmann, C. B. *et al.* Designer Deletion Strains derived from Saccharomyces cerevisiae S288C: a Useful set of Strains and Plasmids for PCR-mediated Gene Disruption and Other Applications. *Yeast* **14**, 115-132 (1998).
