# Supplementary material for: The DEAD-box ATPase Dbp10/DDX54 initiates peptidyl transferase center formation during 60S ribosome biogenesis
Source: Nat Commun. 2024 Apr 17;15:3296. doi: 10.1038/s41467-024-47616-7 (PMC11024185; doi:10.1038/s41467-024-47616-7)
Supplement: Supplementary file 3 — Description of Additional Supplementary Files [file 41467_2024_47616_MOESM3_ESM.pdf]

Description of Additional Supplementary Files

File name: Supplementary Movie 1

Animation of the Dbp10 catalytic cycle. The movie begins with an overall view of the pre-catalysis state, zooming in on the H92 structure and showing its position relative to H61. Next, we show our proposed engagement of H92alt by the Dbp10-D2 domain, followed by ATP-binding dependent unwinding of H92alt by the assembly of a functional Dbp10-D1/D2 interface. H92alt unwinding leads to extensive rRNA remodeling and formation of the A-loop and the H90/H91/H92 three-way junction. After substrate release, Dbp10 assumes a post-catalysis state, which is stabilized by the Dbp10-CTT (yellow). In this conformation, Dbp10 engages H61 (green) and H64 (orange) with additional tail elements (grey and magenta). H61/H64 rearrangement in turn promotes the release of Rrp14 (red), which allows the C-terminal domain of Spb1 to bind (brown). Sbp1 is hub protein that guides Noc2/Noc3 (red/blue) binding to promote the docking of rRNA domain III onto the pre-60S core in the post-catalysis state, shown at the end of the animation.
