# Supplementary material for: The DEAD-box ATPase Dbp10/DDX54 initiates peptidyl transferase center formation during 60S ribosome biogenesis
Source: Nat Commun. 2024 Apr 17;15:3296. doi: 10.1038/s41467-024-47616-7 (PMC11024185; doi:10.1038/s41467-024-47616-7)
Supplement: Supplementary file 5 — Reporting Summary [file 41467_2024_47616_MOESM5_ESM.pdf]

nature portfolio

Corresponding author(s): Jan P. Erzberger

Last updated by author(s): 2024/03/25

Reporting Summary

Nature Portfolio wishes to improve the reproducibility of the work that we publish. This form provides structure for consistency and transparency in reporting. For further information on Nature Portfolio policies, see our Editorial Policies and the Editorial Policy Checklist.

Statistics

For all statistical analyses, confirm that the following items are present in the figure legend, table legend, main text, or Methods section.

n/a	Confirmed	
☒	☐	The exact sample size (*n*) for each experimental group/condition, given as a discrete number and unit of measurement
☐	☒	A statement on whether measurements were taken from distinct samples or whether the same sample was measured repeatedly
☒	☐	The statistical test(s) used AND whether they are one- or two-sided<br>*Only common tests should be described solely by name; describe more complex techniques in the Methods section.*
☒	☐	A description of all covariates tested
☒	☐	A description of any assumptions or corrections, such as tests of normality and adjustment for multiple comparisons
☐	☒	A full description of the statistical parameters including central tendency (e.g. means) or other basic estimates (e.g. regression coefficient) AND variation (e.g. standard deviation) or associated estimates of uncertainty (e.g. confidence intervals)
☒	☐	For null hypothesis testing, the test statistic (e.g. *F*, *t*, *r*) with confidence intervals, effect sizes, degrees of freedom and *P* value noted<br>*Give P values as exact values whenever suitable.*
☒	☐	For Bayesian analysis, information on the choice of priors and Markov chain Monte Carlo settings
☒	☐	For hierarchical and complex designs, identification of the appropriate level for tests and full reporting of outcomes
☒	☐	Estimates of effect sizes (e.g. Cohen's *d*, Pearson's *r*), indicating how they were calculated

Our web collection on statistics for biologists contains articles on many of the points above.

Software and code

Policy information about availability of computer code

Data collection	Cryo-EM data collection: Serial EM 3.7.11
Data analysis	Cryo-EM data analysis: MotionCorr2, GCTF, Phenix v1.19.1,<br>Model building: Coot v 0.8.9.2<br>Structure Refinement: RELION 4.1.<br>Molecular Graphics: CHIMERA X, Pymol<br>Mass spectrometry analysis: Proteome Discoverer 2.4

For manuscripts utilizing custom algorithms or software that are central to the research but not yet described in published literature, software must be made available to editors and reviewers. We strongly encourage code deposition in a community repository (e.g. GitHub). See the Nature Portfolio guidelines for submitting code & software for further information.

Data

Policy information about availability of data

All manuscripts must include a data availability statement. This statement should provide the following information, where applicable:

- Accession codes, unique identifiers, or web links for publicly available datasets
- A description of any restrictions on data availability
- For clinical datasets or third party data, please ensure that the statement adheres to our policy

The cryo-EM density maps and models have been deposited in EMDB and PDB databases under accession codes: EMD-43017/PDB-8V83 (pre-catalytic structure – overall map and model), EMD-43018 (pre-catalytic structure – Local map PTC focused), EMD-43019 (pre-catalytic structure – Local map L1 focused), EMD-43020 (pre-catalytic structure – Local map Rrp14/Rrp15/Ssf1 focused), EMD-43021/PDB-8V84 (catalytic structure – overall map and model), EMD-43022 (catalytic structure – Local map Dbp10 focused), EMD-43024 (catalytic structure – Local map L1 focused), EMD-43026 (catalytic structure – Local map Rrp14/Rrp15/Ssf1 focused), EMD-43023/PDB-8V85 (catalytic structure – 6Å low-pass-filtered, locally-refined map and docked D1 model), EMD-43027/PDB-8V87 (post-catalytic structure – overall map and model), EMD-43028 (post-catalytic structure – Local map Dbp10 focused), EMD-43029 (post-catalytic structure – Local map H61/H64 focused). Strain and plasmid information, cryo-EM micrographs, 2D classes, FSC curves, ResMap plots, and data processing 3D-classification and particle sorting schemes generated in this study are provided in the Supplementary Information/Source Data file.

Research involving human participants, their data, or biological material

Policy information about studies with human participants or human data. See also policy information about sex, gender (identity/presentation), and sexual orientation and race, ethnicity and racism.

Reporting on sex and gender	There were no human participants in this study
Reporting on race, ethnicity, or other socially relevant groupings	There were no human participants in this study
Population characteristics	There were no human participants in this study
Recruitment	There were no human participants in this study
Ethics oversight	There were no human participants in this study

Note that full information on the approval of the study protocol must also be provided in the manuscript.

Field-specific reporting

Please select the one below that is the best fit for your research. If you are not sure, read the appropriate sections before making your selection.

☒ Life sciences ☐ Behavioural & social sciences ☐ Ecological, evolutionary & environmental sciences

For a reference copy of the document with all sections, see nature.com/documents/nr-reporting-summary-flat.pdf

Life sciences study design

All studies must disclose on these points even when the disclosure is negative.

Sample size	The number of particles chosen for our cryo-EM reconstructions are not predetermined but are of sufficient quantity to enable a high-resolution reconstruction. No mathematical sample size calculation was performed. Detailed information about sample size for each of our datasets, and technical or biological replicates are provided in Figure Legends or Extended Data.
Data exclusions	The particle selection strategy for our cryo-EM reconstructions are described in Extended Data, micrographs with poor Thon rings, devitrified ice, contamination from debris or extreme drift were discarded. For yeast growth experiments no relevant datasets have been excluded.
Replication	All genetic experiments were repeated at least three times with comparable results. All plasmid sequences and strains were confirmed by sequencing. Purification of pre-ribosomes was performed 2-5 times with comparable results.
Randomization	Particles are randomized and split into two datasets for calculation of Fourier Shell Correlation curves during cryo-EM resolution estimation in Relion 3.1. Purification of pre-ribosomes, genetic experiments comparing growth of different mutant strains and microscopy experiments randomization all rely on standard assays that do not require any randomization.
Blinding	Purification of pre-ribosomes, grid preparation and data collection was performed by the same investigator using the same parameters for all samples. Yeast growth assays were also performed by the same investigator and did not require subjective interpretation of the results. All fluorescent microscopy was performed by the same investigator using similar parameters. Because no subjective interpretation of results was needed in this study blinding is not applicable.

Reporting for specific materials, systems and methods

We require information from authors about some types of materials, experimental systems and methods used in many studies. Here, indicate whether each material, system or method listed is relevant to your study. If you are not sure if a list item applies to your research, read the appropriate section before selecting a response.

Materials & experimental systems

n/a	Involved in the study
☒	☐ Antibodies
☒	☐ Eukaryotic cell lines
☒	☐ Palaeontology and archaeology
☒	☐ Animals and other organisms
☒	☐ Clinical data
☒	☐ Dual use research of concern
☒	☐ Plants

Methods

n/a	Involved in the study
☒	☐ ChIP-seq
☒	☐ Flow cytometry
☒	☐ MRI-based neuroimaging

Plants

Seed stocks	No seed stocks were used in this study
Novel plant genotypes	None
Authentication	No plants were used in this study
